# Downregulation of TrkC Receptors Increases Dendritic Arborization of Purkinje Cells in the Developing Cerebellum of the Opossum, *Monodelphis domestica*

**DOI:** 10.3389/fnana.2020.00056

**Published:** 2020-09-10

**Authors:** Beata Tepper, Katarzyna Bartkowska, Malgorzata Okrasa, Sonia Ngati, Magdalena Braszak, Krzysztof Turlejski, Ruzanna Djavadian

**Affiliations:** ^1^Laboratory of Calcium Binding Proteins, Nencki Institute of Experimental Biology Polish Academy of Sciences, Warsaw, Poland; ^2^Faculty of Biology and Environmental Sciences, Cardinal Stefan Wyszynski University in Warsaw, Warsaw, Poland

**Keywords:** development, cerebellum, TrkC receptor, shRNA, BrdU, *Monodelphis*, opossum

## Abstract

In therian mammals, the cerebellum is one of the late developing structures in the brain. Specifically, the proliferation of cerebellar granule cells occurs after birth, and even in humans, the generation of these cells continues during the first year of life. The main difference between marsupials and eutherians is that the majority of the brain structures in marsupials develop after birth. Herein, we report that in the newborn laboratory opossum (*Monodelphis domestica*), the cerebellar primordium is distinguishable in Nissl-stained sections. Additionally, bromodeoxyuridine birthdating experiments revealed that the first neurons form the deep cerebellar nuclei (DCN) and Purkinje cells, and are generated within postnatal days (P) 1 and 5. Three weeks after birth, progenitors of granule cells in the external germinal layer (EGL) proliferate, producing granule cells. These progenitor cells persist for a long time, approximately 5 months. Furthermore, to study the effects of neurotrophic tropomyosin receptor kinase C (TrkC) during cerebellar development, cells were obtained from P3 opossums and cultured for 8 days. We found that *TrkC* downregulation stimulates dendritic branching of Purkinje neurons, which was surprising. The number of dendritic branches was higher in Purkinje cells transfected with the shRNA *TrkC* plasmid. However, there was no morphological change in the number of dendritic branches of granule cells transfected with either control or shRNA *TrkC* plasmids. We suggest that inhibition of TrkC activity enables NT3 binding to the neurotrophic receptor p75^NTR^ that promotes dendritic arborization of Purkinje cells. This effect of TrkC receptors on dendritic branching is cell type specific, which could be explained by the strong expression of TrkC in Purkinje cells but not in granule cells. The data indicate a new role for TrkC receptors in *Monodelphis* opossum.

## Introduction

In all mammalian species including marsupials, the cerebellum contains two hemispheres connected with a medially located structure, the vermis, and the laterally located flocculus and paraflocculus. The main types of neurons, Purkinje cells and granule cells, and their projections are regularly arranged, forming a laminar organization of the cerebellum (Cajal, 1960). The Purkinje cell is one of the largest neurons in the brain. It has a large cell body and a unique and distinguishable dendritic tree that produces a γ-aminobutyric acid (GABA) inhibitory neurotransmitter, while granule cells have a small somata and produce glutamate, an excitatory neurotransmitter (for review see Gill and Sillitoe, 2019). In rats, neurons of the deep nuclei and Purkinje cells are generated in the ventricular zone on embryonic day (E) 14 and E15, and granule cells proliferate from the caudal part of the ventricular zone known as the rhombic lip (Altman and Bayer, 1985a,b,c; Hausmann et al., 1985). Progenitor cells that proliferate in the rhombic lip migrate and create the second germinal zone called the external germinal layer (EGL). The EGL produces progenitor cells that migrate in the inner granular layer (IGL) and differentiate into neurons (Rakic and Sidman, 1970; Hatten and Heintz, 1995; Alder et al., 1996; Weisheit et al., 2006). In rats, the proliferation of cerebellar interneurons and granule cells that arise from the EGL lasts for over 3 weeks after birth.

Neurotrophins and their tropomyosin receptor kinase (Trk) are implicated in the development of neurons and regulate different developmental stages (for review see Bartkowska et al., 2010; Bothwell, 2014). It has been reported that brain-derived neurotrophic factor (BDNF) regulates the early stages of development of cerebellar granule cells, while neurotrophin 3 (NT3) regulates maturation of these cells (Segal et al., 1992). Cerebellar granule cells from the EGL, where granule cells proliferate, migrate to their final destination, the internal granule cell layer (IGL). This migration process is impaired in BDNF-deficient mice (Borghesani et al., 2002). BDNF deficiency in mice causes abnormal development of Purkinje cells (Schwartz et al., 1997). TrkC receptors are also required for the normal development of Purkinje cells. Disturbances of the TrkC/NT3 signaling pathway affect the dendritic arborization process of Purkinje cells (Joo et al., 2014). NT3 also binds and activates another receptor, the neurotrophin receptor p75 (p75^NTR^) that is widely expressed in the developing brain, including the cerebellum (Dechant et al., 1997; Carter et al., 2003).

The opossum is an excellent animal model for studying brain development. Opossums are born after 14 days of gestation with an immature brain, and most processes of brain development take place after birth (Saunders et al., 1989; Brunjes et al., 1992; Cheung et al., 2010). The nervous system of a newborn opossum is at a developmental stage comparable to that on embryonic day (E) 12 in a rat or that of a 40-day-old human embryo (Saunders et al., 1989; Molnár et al., 1998). Our previous studies have shown that downregulation of TrkC and TrkB signaling by shRNA constructs transfected in cultured progenitor cells mainly regulates the proliferation of cortical cells (Bartkowska et al., 2013, 2014). Additionally, *in vivo* electroporation experiments with shRNA *TrkC* and *TrkB* constructs revealed that the blockage of TrkC and TrkB signaling in neocortical progenitor cells affects migration processes, leading to the arrest of neuroblasts at the base of the subplate for a short time (Bartkowska et al., 2019). Thus, the next question we are interested in is whether the role of TrkC receptors is specific to the neocortex and whether they are also involved in the development of other brain structures.

The aim of this study was to investigate the specific morphological types of cells generated during the development of the opossum cerebellum. First, we studied the development of the cerebellum using specific molecular markers known for each type of cerebellar cell from eutherian species studies, and we then examined the birthdate of Purkinje cells and other cell types in the cerebellum of opossums. Further, we asked whether the downregulation of TrkC receptors affects cerebellar developmental events, particularly the development of Purkinje cells and granule cell morphology.

## Materials and Methods

### Animals

Opossums, *Monodelphis domestica*, of both sexes bred at the Nencki Institute of Experimental Biology were used. All animals had *ad libitum* access to water and food. The housing facility was maintained at appropriate temperature (26–28°C) and humidity (50%–70%) and on a regulated daily cycle of 14/10 h (day/night). All efforts were made to minimize the number of animals used and the level of stress they endured. The experimental procedures complied with the Polish Law on Experiments on Animals, which implements the European Council Directive, and were approved by the Local Ethics Committee in Warsaw.

### Animal Treatment and Tissue Preparation

**Figure 1:**
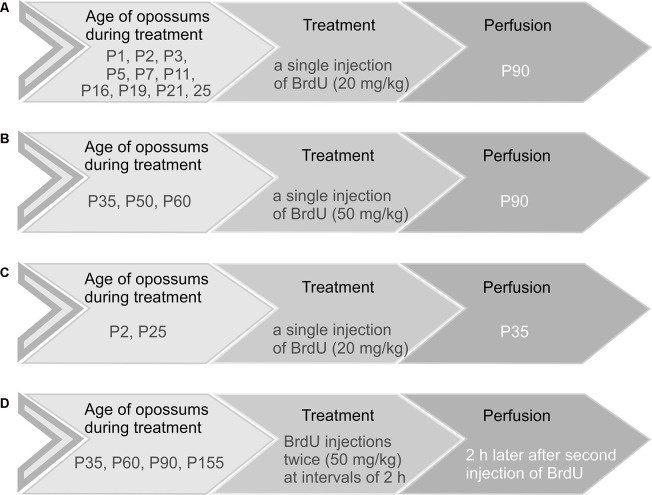
Schematic representation of the four main groups of opossums injected with BrdU. **(A,C)** Opossums of different ages were administered 20 mg/kg body weight of BrdU and perfused at P90 **(A)** or P35 **(C)**. **(B)** Opossums at P35, P50, and P60 were treated with a single dose of BrdU (50 mg/kg) and perfused at P90. **(D)** Opossums at P35, P60, P90, and P155 were injected with BrdU twice (50 mg/kg). Two hours later, after the second injection of BrdU, opossums were perfused. Each age group consisted of 3–6 opossums.

Opossums at the ages of 1, 2, 3, 5, 7, 11, 16, 19, 21, and 25 days received a single injection of BrdU (Sigma–Aldrich) at a dose of 20 mg/kg (Figure 1A). Opossums at P35, P50, and P60 were injected with 50 mg/kg BrdU (Figure 1B). These animals were perfused at the age of 3 months (P90) with saline followed by 4% paraformaldehyde in 0.1 M phosphate buffer (pH 7.4). In two groups, three out of six opossums that were injected with BrdU on P2 and P25 were perfused on P35 (Figure 1C). To study the proliferation of granule cells in the EGL, opossums at P35, P60, P90, and P155 were injected with 50 mg/kg BrdU twice at 2-h intervals and were perfused 2 h later after the last BrdU injection (Figure 1D). Each group consisted of 3–6 opossums. The brains were removed, post-fixed in 4% paraformaldehyde solution, and cut into 40-μm coronal sections in a cryostat. The brain sections were arranged in a series of 10.

### BrdU Immunohistochemistry

Immunohistochemical staining for BrdU was performed on free-floating brain sections. To block endogenous peroxidases, the sections were soaked for 30 min in 3% H_2_O_2_ and 10% methanol in Tris-buffered saline (TBS). Afterwards, the sections were rinsed for 15 min in TBS with 0.1% Triton X-100 (TBS-A) and 15 min in TBS-A with 0.05% bovine serum albumin (TBS-B). The sections were denatured in 2 M HCl at 37°C for 30 min and left in 0.1 M H_3_BO_3_ for 10 min at 22°C. After rinsing in TBS, TBS-A, and TBS-B for 10 min each, the sections were incubated in 10% normal goat serum (NGS) solution in TBS-B for 60 min, and then incubated overnight at 4°C with rat anti-BrdU monoclonal primary antibody (1:500, Santa Cruz) in TBS-B. After a 15-min wash with TBS-A and TBS-B, the sections were incubated for 60 min with a biotinylated goat secondary antibody (1:200, Jackson ImmunoResearch) in TBS-B. The sections were rinsed with TBS-A three times for 5 min and left for 60 min in ExtrAvidin (1:400, Sigma) diluted in TBS. After rinsing, the slices were incubated with diaminobenzidine enhanced with nickel salts (DAB Substrate Kit, Vector), rinsed again, placed on microscope slides, and mounted with DePeX mounting medium (Serva).

Double immunofluorescent staining was performed as described above, but one of the primary antibodies, namely, calbindin (1:1,000, Chemicon), NeuN (1:50, Cell Signaling), GFAP (1:500, Dako), and Olig2 (1:100, Millipore, Kankakee, IL, USA), was added to the solution with rat anti-BrdU primary antibody. The appropriate fluorochrome-conjugated antibodies were selected: goat anti-rabbit 568 (1:500, Abcam), chicken anti-goat 488 (1:500, AlexaFluor Invitrogen), goat anti-mouse 568 (1:500, AlexaFluor Invitrogen), and biotinylated goat secondary antibody (1:200, Jackson ImmunoResearch) followed by fluorochrome-conjugated streptavidin (1:500, AlexaFluor Invitrogen). The brain sections were mounted on slides and cover-slipped with a mounting medium (65% glycerol in PBS).

### Nissl Staining

The brains of opossums at P1, P2, P3, P5, P9, P11, P16, P21, P25, and P35 were immersed in 4% paraformaldehyde for 2 weeks and subsequently cut into 20-μm coronal sections in a cryostat. Each group consisted of 3–5 opossums. Slides with tissue sections were immersed for 20 min in a mixture of chloroform and ethyl alcohol (1:1) for degreasing. Brain sections were gradually hydrated by immersion in a series of decreasing concentrations of ethanol (100%, 96%, 70%, and 50%) and in distilled water for 2 min. Afterwards, the slides were transferred to a solution of cresyl violet in acetic buffer (0.34 M acetic acid; 0.06 M sodium acetate, pH 3.6) for approximately 5 min. The next steps included incubation in distilled water for 2 min, incubation in 50% ethanol for 2 min, and incubation in a solution of 70% ethanol with 10% acetic acid for approximately 30 s. The slides were dehydrated in solutions with increasing ethanol concentrations of 96% and 100% for 2 min. The stained sections were successively immersed in ethanol mixtures with the addition of xylene (1/3 xylene and 2/3 ethanol, 2/3 xylene and 1/3 ethanol, and 100% xylene). The slides were cover-slipped with the mounting medium DePeX (Serva).

### Immunofluorescent Labeling

Immunofluorescent labeling on opossum brains from P1 to P25 was performed on slides with brain sections, while at P35, free-floating brain sections were used. Brain sections were incubated for 1 h with either 10% NGS, 10% normal chicken serum (Sigma–Aldrich), or 1% bovine serum albumin in PBS. After, the sections were incubated overnight in rabbit anti-calbindin (1:1,000, Chemicon), rabbit anti-NeuN (1:50, Cell Signaling), mouse anti-Olig2 (1:100, Millipore, Kankakee, IL, USA), rabbit anti-Tbr1 (1:200, Abcam), rabbit anti-GFAP (1:500, Dako), rabbit anti-TrkC (1:500, Cell Signaling), and rabbit anti-NT3 (1:300, Alomone Labs) primary antibodies. The appropriate secondary antibodies, namely, goat anti-rabbit 568, chicken anti-goat 488, and goat anti-mouse 488 were used. Finally, the sections were mounted on slides and cover-slipped with a 60% glycerol solution in PBS.

### Western Blot

Opossums at P4, P24, and P35 were euthanized. Their brains were isolated, and the different structures were separated on ice. The cerebellums were collected. The cerebellar tissue lysates were prepared from the pooled cerebellums of 5 to 6 opossums at P4. For older ages, at P24 and P35, lysates containing the cerebellum from each animal were used. They were mechanically homogenized in lysis buffer with protease inhibitors (Roche), treated with detergents NP 40 (Fluka) and sodium dodecyl sulfate (SDS, Sigma), and incubated for 15 min. Afterwards, they were centrifuged at 14,000 rpm for 45 min at 4°C. The supernatant was collected, aliquoted, and stored at −70°C. Protein samples (30 μg/lane) were loaded on a 7.5% SDS-PAGE and electroblotted onto a nitrocellulose membrane for 2 h at 390 mA at 4°C. The blots were incubated in 5% skimmed milk powder dissolved in TBS with 0.2% Tween 20 for 1 h 30 min and incubated overnight at 4°C with rabbit anti-TrkC (1:1,200, C44H5 Cell Signaling) or rabbit anti-p75^NTR^ (1:1,000, Abcam). The blots were incubated with goat anti-rabbit secondary antibody conjugated with horseradish peroxidase (1:2,000, Bio-Rad Laboratories). For visualization of the TrkC protein, an enhanced chemiluminescence solution was applied.

As a loading control, GAPDH protein was used. To remove the TrkC bound protein from blots, a Re-Blot Plus Stripping Solution was used. After blocking, the blots were incubated with mouse anti-GAPDH antibody (1:10,000, Millipore, Kankakee, IL, USA) and goat anti-mouse secondary antibody (1:7,000, Chemicon).

### Cell Cultures

Primary cerebellar cell cultures were obtained by isolating cerebellums from 3-day-old opossum pups for Purkinje cells and from 22-day-old pups for granule cells. Cells were plated on 2-well chamber slides (BIOLOGIX group) covered with poly-L-lysine and laminin (Sigma–Aldrich) at a seeding density of 3 × 10^5^ cells in 1.5 ml of medium per well. The culture medium contained 1% B27 50× (Gibco), 2 mM Glutamax (Gibco), 1% penicillin/streptomycin, 0.45% glucose, 3 mM KCl, 1% HEPES, 1% FBS, and 400 ng FGF-2 (Sigma–Aldrich) dissolved in Neurobasal A (Gibco). After 24 h, cells were transfected with shRNA TrkC or shRNA control plasmids expressing GFP using Lipofectamine 3000 transfection reagent (Thermo Fisher Scientific). The sequence for shRNA TrkC was 5′-CACCGCAGTAAGACAGAGATCAATTTCAAGAGAATTGATCTCTGTCTTACTGCTTTTTTG-3′. The control shRNA was mismatched to known human and mouse genes (EZBiolab).

On the 8th or the 10th day *in vitro* (DIV), cells cultures were fixed in 4% paraformaldehyde solution for 10 min, washed in PBS, and incubated with 0.1% NP-40 detergent (Sigma–Aldrich). The slides were rinsed with PBS, incubated with the blocking solution (10% NGS and 1% bovine serum albumin in PBS), and incubated overnight at 4°C with primary antibodies: mouse anti-GFP (1:750, Sigma–Aldrich), rabbit anti-GFP (1:750, Millipore, Kankakee, IL, USA), chicken anti-MAP2 (1:200, Abcam), rabbit anti-calbindin (1:1,000, Chemicon), mouse anti-calbindin (1:2,000, Swant), rabbit anti-NT3 (1:300, Alomone Labs), and rabbit anti-TUJ (1:750, Covance) solution in PBS. The next day, the slides were rinsed with PBS and incubated with secondary antibodies (goat anti-mouse 488, goat anti-rabbit 488, goat anti-chicken 568, and goat anti-rabbit 568) diluted in PBS for 1 h at room temperature. After rinsing, the slides were incubated with DAPI (1:5,000, Invitrogen) solution in PBS for 7 min, rinsed again, mounted in 65% glycerol in PBS, and closed with coverslips.

### Data Analysis and Statistics

Images from Nissl-stained tissues were obtained using a Nikon Eclipse 90i microscope connected to a computer with Image Pro software. Pictures from immunofluorescent sections and cell cultures were captured and analyzed using a confocal laser microscope (Zeiss). Double-immunolabeled GFP and CB neurons were traced and analyzed using Neurolucida software (MBF Bioscience). Primary dendrites were counted, and the complexity of dendritic branching of Purkinje neurons was determined using Sholl analysis with 10-μm intervals between concentric circles. Morphological quantification of granule cells was performed using Neurolucida from images taken with a Zeiss confocal laser scanning microscope. Differences in the number of primary dendrites, length, and intersections of all dendrites in shRNA TrkC and shRNA control neurons were tested using Student’s *t* test (two-tailed, independent samples, equal variances) in GraphPad Prism. Differences were considered significant at *p* < 0.05.

## Results

### Development of the Cerebellum

**Figure 2:**
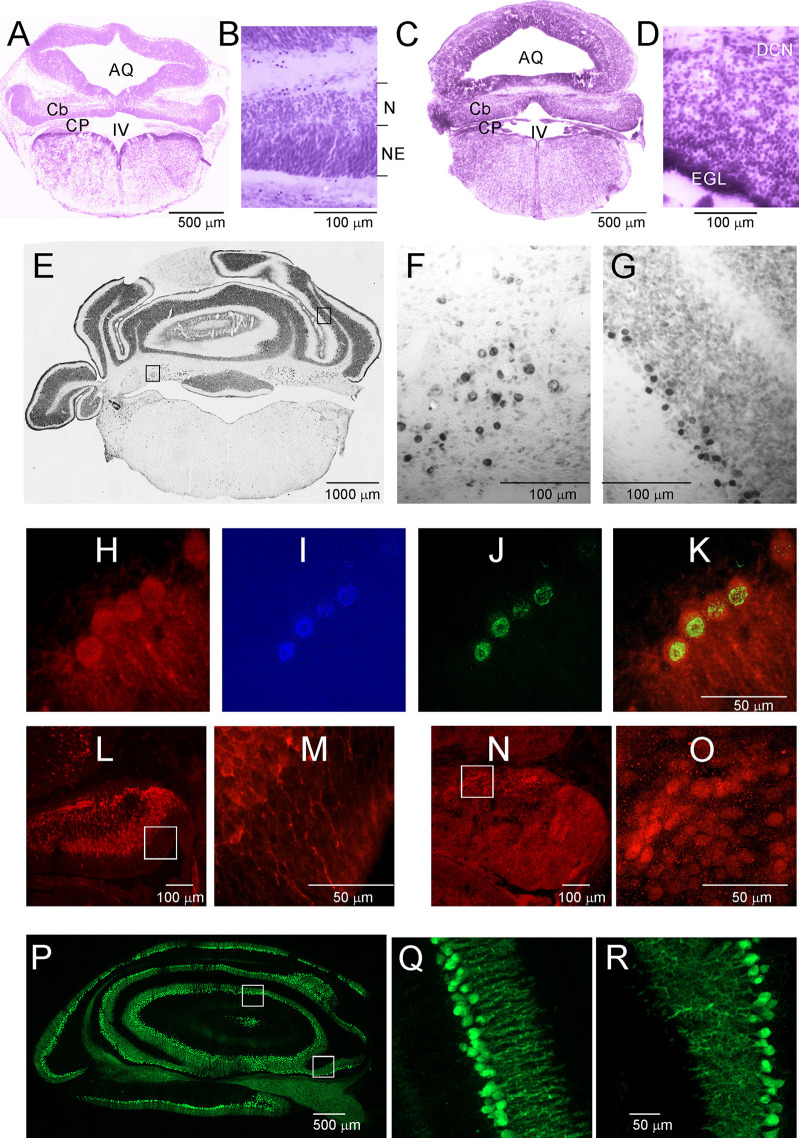
Postnatal development of the cerebellum in the *Monodelphis* opossum. **(A–D)** Nissl-stained sections in the cerebellum of 2-day-old **(A,B)** and 5-day-old **(C,D)** opossums. **(E)** A coronal section at the level of the cerebellum in an opossum that was injected with BrdU at P1. High-magnification images showing cells of the DCN **(F)** and Purkinje cells **(G)** generated on P1 labeled with BrdU. **(H–K)** Cells labeled with CB **(H)**, DAPI **(I)**, BrdU **(J)**, and merged image of the three labels **(K)**. **(L,M)** CB-labeled cells in the cerebellum of a 3-day-old opossum. **(N,O)** Tbr1-labeled cells in the cerebellum of a 3-day-old opossum. **(P–R)** CB labeling in the cerebellum of a 35-day-old opossum. IV, fourth ventricle; AQ, aqueduct of Sylvius; Cb, cerebellum; CB, calbindin; CP, choroid plexus; DCN, deep cerebellar nuclei; EGL, external germinal layer; N, neurons; NE, neuroepithelium.

Opossums are born at a very early stage of development. The newborn opossum, with a body weight of 117.5 mg (±8.0), is similar to a 13-day-old rat embryo (Supplementary Figure S1). The forelimbs of the newborn opossum are more developed than the hindlimbs, and several brain structures, including the cerebellum, would not have developed at that point. On P2, the region that seems to develop in the caudal part of the brain is the brainstem (Figure 2A). Other structures were at an early stage of development, mainly consisting of progenitor cells. During this period, the cerebellar primordium is distinguishable. High-magnification Nissl-stained images of this brain region showed the presence of two types of cells: progenitor cells and neurons (Figure 2B). Progenitor cells were characterized by prolonged cell soma and stained darker than neurons (Figure 2B). To study the generation and migration of cells that form the cerebellum, opossums at P1 were injected with BrdU, while BrdU-labeled cells were examined 1 month or 3 months later. The vast majority of BrdU-positive cells that were generated on P1 migrated and were located in both the deep cerebellar nuclei (DCN) of the cerebellum (Figures 2E,F) and the Purkinje cell layer (Figures 2E,G).

Five-day-old opossums markedly grew and developed (Supplementary Figure S1). The body weight was 208 ± 8.3 g. The cerebellar plate enlarged its territory, but this structure was still undifferentiated (Figure 2C). In the cerebellar primordium, there were more neurons than progenitor cells, as can be seen in the Nissl-stained sections (Figure 2D). At P5, a new layer of EGL appeared in the cerebellar primordium (Figures 2C,D). Some transcription factors are specifically expressed in neurons during the formation of the cerebellum. We characterized neuron types using molecular markers including the T-box brain transcription factor 1 (*Tbr1*), basic helix-loop-helix (bHLH) oligodendrocyte transcription factor *Olig2*, and Calbindin (CB), which was used as a reliable marker for Purkinje cells. During development, *Olig2* is expressed not only in oligodendrocytes but also in neurons. All BrdU-labeled cells, which are Purkinje cells, colocalized with CB (Figures 2H–K). We detected both CB (Figures 2L,M) and Tbr1 (Figures 2N,O) immunoreactive cells that displayed different patterns of distribution in the cerebellum of P3 opossums. Generally, CB was expressed in Purkinje cells, while Tbr1 was expressed in the neurons of the DCN (Figures 2L–O). The intensity of CB staining in Purkinje cells was gradually increased during development of the cerebellum. At P35, developed Purkinje cells were extremely expressed CB (Figures 2P–R).

**Figure 3:**
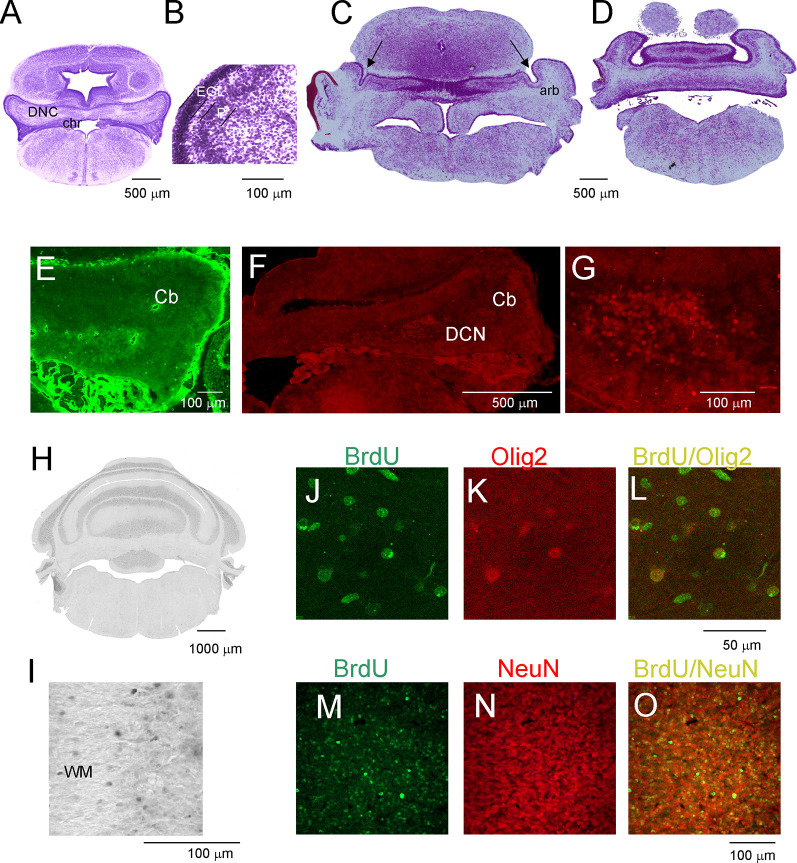
Postnatal development of the cerebellum in the opossum. **(A–D)** Nissl-stained coronal sections on P11 **(A,B)** and P19 **(C,D)** opossum brains. **(E)** Olig2-labeled cells in the cerebellum of an 11-day-old opossum. **(F,G)** Tbr1-labeled cells in the cerebellum of an 11-day-old opossum. **(H,I)** BrdU-labeling pattern in the cerebellum of a 3-month-old opossum that was injected with BrdU at P19. **(J–L)** Double labeling for BrdU (green), Olig2 (red), and merged image of BrdU/Olig2 in the cerebellum of an opossum that was injected with BrdU at P25. **(M–O)** High-magnification images of double labeling for BrdU (green) and GFAP (red) in the cerebellum of an opossum that was treated with BrdU on P25. arb, arbor vitae; CP, choroid plexus; DCN, deep cerebellar nuclei; EGL, external germinal layer; P, Purkinje cells.

The development of the cerebellum was not remarkably progressed between P7 and P16. During this period, expansion in the cerebellum territory was the result of cell growth, but not the increase in their number. The rate of proliferation decreased compared to that in the first week after birth, and the number of generated cells was low if BrdU injections were given at P7 to P16 opossums. At P11, the folding of the EGL was slightly initiated (Figure 3A), but none of the lobules were created in either the vermal or hemispheric segments of the cerebellum. Cytoarchitectonic lamination of the cerebellum was also apparent. The Purkinje cells formed multiple layers. These cells had large cell bodies (Figure 3B) and unbranched dendritic trees (not shown). Intensive labeling of neurons was seen in the DCN using specific antibodies (Figures 3E–G). Tbr1-labeled neurons (Figures 3F,G) and Olig2-labeled neurons (Figure 3E) were observed in the DCN. The same Olig2 marker was used to label oligodendrocytes in older brains. The first oligodendrocytes were originated at P16, and their number increased the next few days (Figures 3J–L).

**(M)** Double-labeled BrdU/NeuN cells in the cerebellum of P90 opossum that was injected with BrdU at P25.

During the subsequent 2 weeks, there appeared to be foliation of the cerebellum. First, the fissures that separated the cerebellar vermis and hemispheres were generated, and additional fissures subdivided the vermis and hemispheres into lobules. At P19, these fissures that separated the vermis from the hemispheres were evident (Figures 3C,D). In addition, the branched white matter called arbor vitae (arb) was apparent (Figure 3C). The generation of the fissure was concomitant with the generation of granule cells. Numerous BrdU-labeled cells were observed in 3-month-old opossums that were injected at P19 (Figures 2H,I). The number of BrdU cells was increased in the brains of opossums that received BrdU injections at P25. The vast majority of BrdU-labeled cells were neurons. This was confirmed using double labeling for BrdU and NeuN (Figures 3M–O).

**Figure 4:**
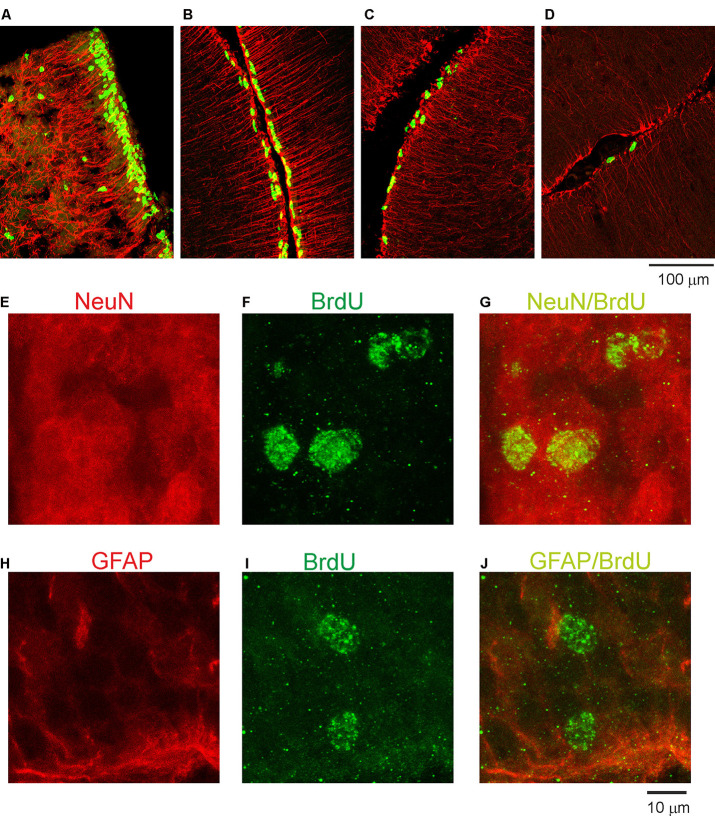
Proliferative activity in the developing cerebellum of the opossum. **(A–D)** Immunocytochemical staining for BrdU (green) and GFAP labeling (red) in the cerebellum of P35 **(A)**, P60 **(B)**, P90 **(C)**, and P155 **(D)** opossums. **(E–G)** High-magnification confocal images for NeuN (red), BrdU (green), and merged NeuN/BrdU labeling in the cerebellum of a 3-month-old opossum that was injected with BrdU on P60. **(H–J)** High-magnification confocal images for GFAP (red), BrdU (green), and merged GFAP/BrdU labeling in the cerebellum of a 3-month-old opossum that was injected with BrdU on P60.

Next, we examined the duration for the development of the second germinal zone (EGL), from which progenitors of granule cells are derived. In the opossum, the EGL appears in the primordial cerebellum on P5; however, the first granule cells are generated at P16 with a peak between P25 and P35. To study this developmental event, we injected opossums with BrdU twice at 2-h intervals at P35, P60, P90, and P155 and perfused them 2 h later. Immunostained BrdU cells indicated that granule progenitor cells were still divided. Bergmann glia cells were detected with GFAP immunostaining (Figures 4A–D). We found that the EGL was quite thick on P35 (Figure 4A); however, its thickness gradually decreased in the following months (Figures 4B,C). At P155, BrdU-immunopositive cells were occasionally observed (Figure 4D), indicating that the proliferation of granule cells was stopped. Almost all progenitor cells gave rise to neurons only. Analysis of double-labeled cells for BrdU and NeuN in opossums that were treated with BrdU at P60 and perfused a month later showed that all BrdU cells colocalized with NeuN (Figures 4E–G). Some adjacent sections were stained for BrdU and GFAP (Figures 4H–J). These images showed that there was no colocalization of BrdU- and GFAP-expressing cells.

### Influence of TrkC on the Development of Dendrites in Cerebellar Cells

**Figure 5:**
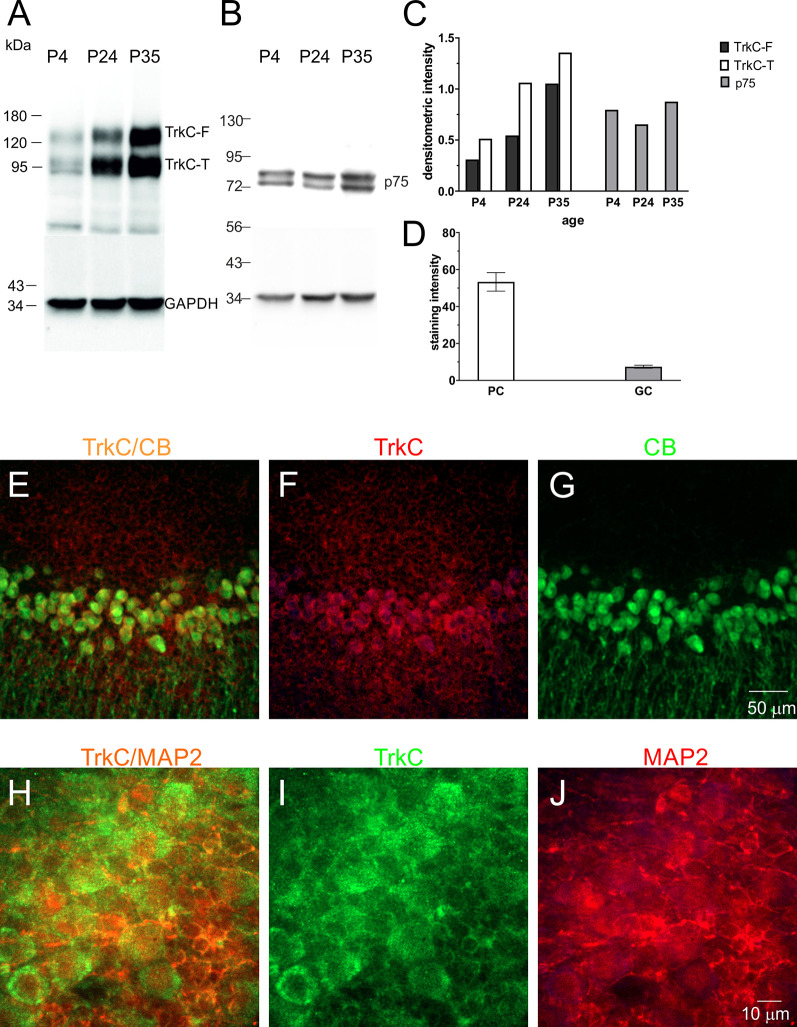
TrkC and p75^NTR^ protein levels in the developing cerebellum of the opossum. Western blot illustrating TrkC-labeled **(A)**, p75^NTR^-labeled **(B)**, and GAPDH-labeled bands in the cerebellum of opossums on P4, P24, and P35. **(C)** Quantitative data from Western blots. TrkC-F, full-length TrkC proteins; TrkC-T, truncated isoform of TrkC receptors. **(D)** TrkC staining intensity in Purkinje and granule cells of the cerebellum. **(E–G)** Double-labeled confocal images for merged TrkC/CB, TrkC (red), and CB (green) in the cerebellum of a 35-day-old opossum. **(H–J)** Double-labeled confocal images for merged TrkC/MAP2, TrkC (green), and MAP2 (red) in the cerebellum of a 35-day-old opossum.

TrkC receptors are expressed in the brains of newborn opossums. The level of this protein increased gradually until P35, the day when opossums open their eyes (Bartkowska et al., 2013). Here, we focused our Western blot analysis on isolated cerebellar tissues. The following developmental time points were selected: P4, which is associated with the generation of Purkinje cells, and P24 and P35, which are associated with the proliferation of granule cells. We found that the level of TrkC receptors was low in the cerebellum at P4 (Figures 5A,C). At P24, the level of TrkC protein was higher than at P4, but the highest level of TrkC was detected at P35 (Figure 5C). We also analyzed the expression of low-affinity neurotrophic receptor p75^NTR^. This receptor was highly expressed in the cerebellar primordium of opossums after birth. Western blot lysates from the cerebellum showed that approximately similar levels of p75^NTR^ were observed at P4 and P35 (Figures 5B,C).

To determine the amount of TrkC in different types of cerebellar cells, we performed a double-immunolabeling technique using TrkC and CB or TrkC and MAP2 markers for identification of Purkinje cells and granule cells, respectively. TrkC was detectable in both Purkinje and granule cells, although TrkC expression was strong in Purkinje cells. The soma and dendrites of Purkinje cells contained high levels of TrkC protein (Figures 5E–G), while only the cell membrane of granule neurons was weakly labeled with TrkC (Figures 5H–J). The quantitative analysis of these data is illustrated in Figure 5D.

**Figure 6:**
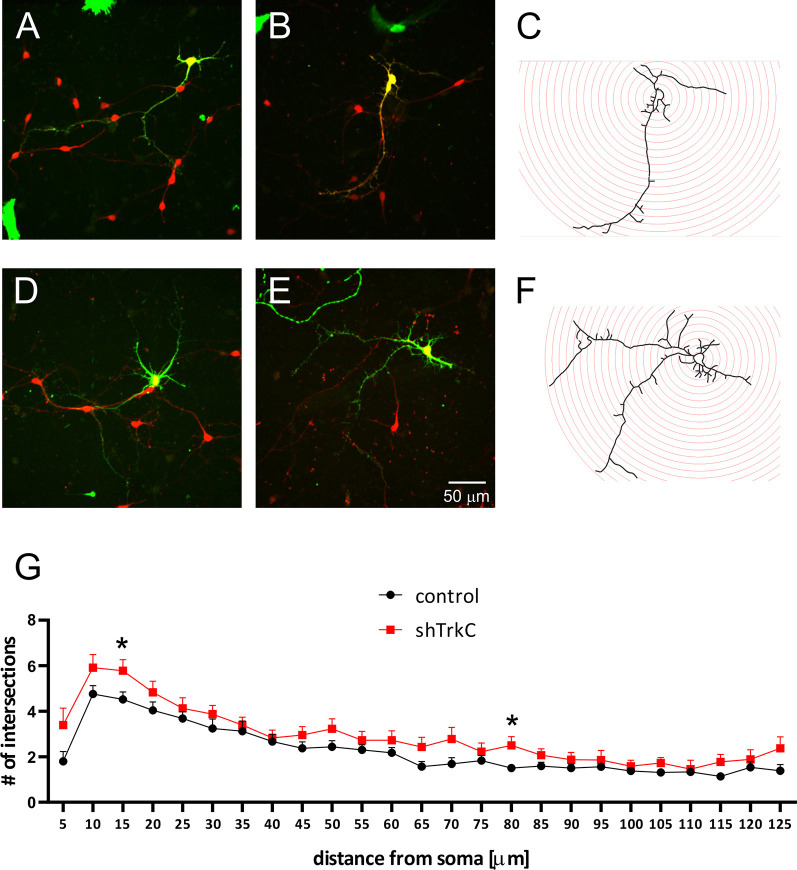
Representative images of Purkinje cells transfected with the control plasmid **(A,B)** and with the shRNA TrkC plasmid **(D,E)**. After 8 days, cultured cells were immunolabeled for GFP (green) and CB (red). Schematic representation of Sholl analysis method for cells that were transfected with shRNA control **(C)** and shRNA *TrkC* **(F)** plasmids. **(G)** The number of dendritic intersections that was calculated at each circle (10-μm intervals between circles). The scale bar on **(E)** refers to **(A–C)**. *p < 0.02.

**Figure 7:**
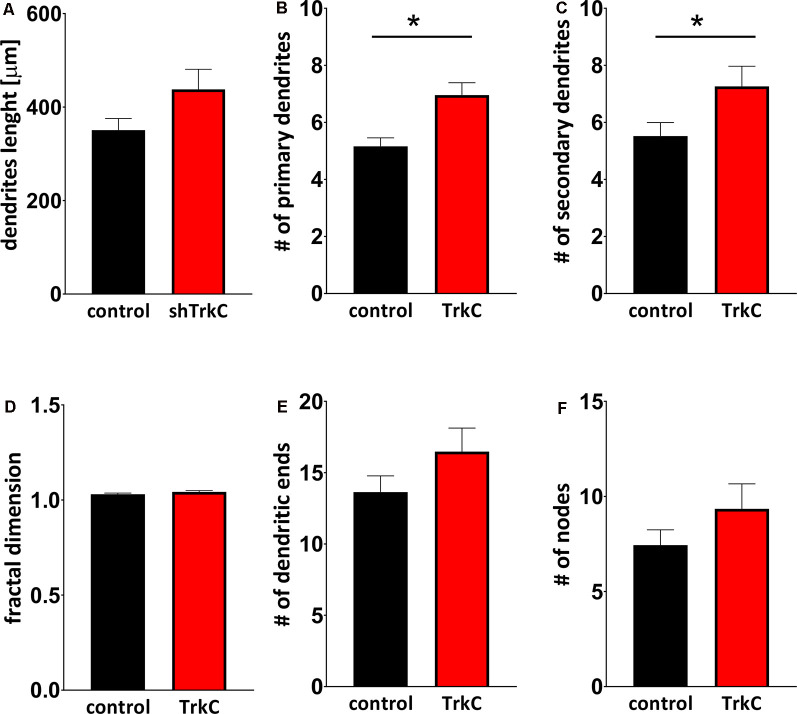
Quantitative analysis of measures describing morphology of dendrites. **(A)** The total length of Purkinje cells. **(B,C)** The number of primary **(B)** and secondary **(C)** dendritic branches. **(D)** Fractal dimension. **(E,F)** The number of dendritic ends **(E)** and nodes **(F)**. In **(B)** *p < 0.001 and in **(C)** *p < 0.04.

Neurotrophins and their receptors are known to enhance the development of neural fibers. To examine the influence of TrkC, a specific receptor for NT3, we constructed shRNA TrkC. Cells were isolated from the cerebellum of P3 opossums, dissociated and transfected with shRNA plasmid, and allowed to grow. Purkinje cell progenitors were cultured from 8 DIV to 10 DIV. We examined only neurons that were co-labeled for GFP and CB, which are supposed to be Purkinje cells. We found that more transfected progenitor cells that proliferated and differentiated into Purkinje cells survived on 8 DIV compared to 10 DIV. Between 7 and 10 DIV, half of the transfected cells died in both cultures transfected with the control plasmid and those transfected with the shRNA TrkC plasmid. A similar result was described in cell cultures of the dissociated mouse cerebellum (Tabata et al., 2000). Therefore, the properties of Purkinje cells were analyzed 8 days after seeding and 7 DIV after transfection. Examples of Purkinje cells with differentiated dendritic trees are shown in Figure 6. We observed that Purkinje cells transfected with shRNA TrkC had more dendritic branches (Figures 6D,E) than cells transfected with the control plasmid (Figures 6A,B). Quantification of the dendritic arborizations of Purkinje cells and their length and number was performed on neurons transfected with control plasmid (*n* = 25) and shRNA TrkC plasmid (*n* = 23) using Sholl analysis in Neurolucida software (Figures 6C,F). We found that the number of dendritic intersections at a distance of 15 μm from the cell body was higher in neurons with downregulated TrkC (Figure 6G). This difference was statistically significant (*p* < 0.02) and referred to the number of primary (*t* = 3.464, *df* = 46, *p* < 0.001) and secondary (*t* = 2.078, *df* = 46, *p* < 0.04) dendrites (Figures 7B,C). Additionally, Sholl analysis revealed that the total length of Purkinje cell dendrites was 350.7 μm (±24.8) in neurons transfected with the control plasmid and 437.8 μm (±42.96) in neurons transfected with the shRNA TrkC plasmid (Figure 7A). However, we found no significant difference (*t* = 1.789, *df* = 46, *p* < 0.08) in the length of neurons between cells, grown in control conditions compared to neurons with downregulated TrkC receptors. Other measures describing morphology of dendrites such as fractal dimension (Figure 7D), the number of dendritic ends (Figure 7E), and the number of nodes (Figure 7F) were applied. None of them showed a statistically significant difference between the two groups.

**Figure 8:**
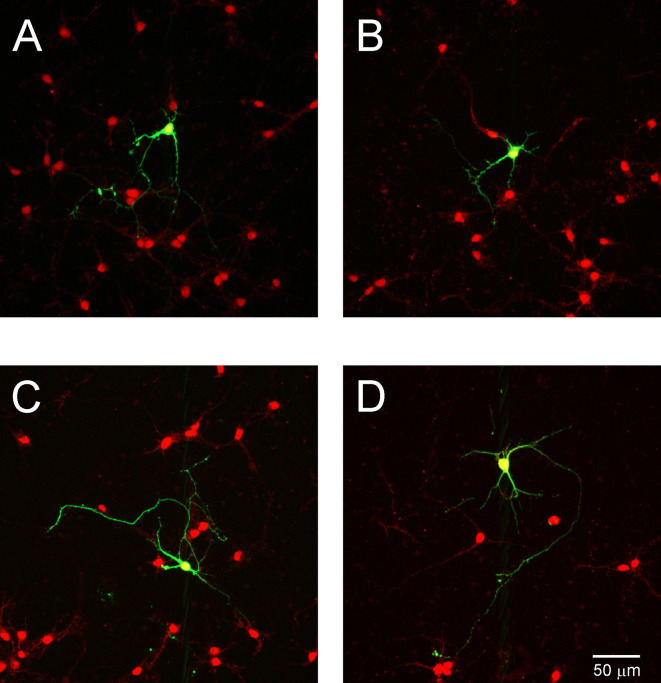
NT3 expression in cell cultures. Representative images of Purkinje cells transfected with the control plasmid **(A,B**) and with shRNA TrkC plasmid **(C,D)**. Cells were immunolabeled for GFP (green) and NT3 (red). The scale bar on **(D)** refers to **(A–C)**.

To determine whether NT3 affects the dendritic development of Purkinje cell, we examined NT3 expression in cell culture. Cells cultured 7 DIV after transfection were stained with anti-NT3 antibody. We observed that numerous neurons were stained with NT3 (Figure 8), indicating that Purkinje cells are grown under condition in which ligand for TrkC receptors is present.

**Figure 9:**
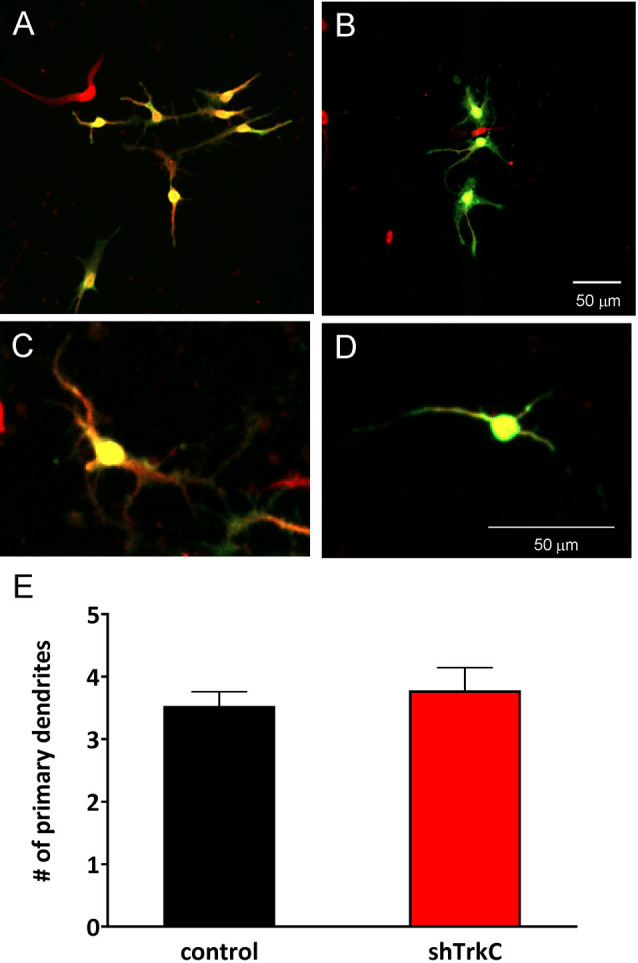
Representative images of granule cells after 7 days in culture that were dissociated from the cerebellum of a 22-day-old opossum. Cells were immunolabeled for GFP (green) and MAP2 (red). **(A,C)** Granule cells transfected with shRNA contro plasmid. **(B,D)** Granule cells transfected with the shRNA *TrkC* plasmid. **(E)** Quantitative analysis of the number of primary dendrites. The scale bar on **(B)** refers to **(A)**, while the scale bar on **(D)** refers to **(C)**.

To assess the effect of TrkC activity inhibition on dendritic branches in granule cells, we dissociated progenitor cells from the cerebellum of opossums on P22. The next day, cells were transfected with the shRNA plasmid and cultured for 7 days, which was sufficient for the development of granule cell processes (Figures 9A–D). Analysis of granule cell arborization was carried out on 18–19 cells from each group. The data revealed that the dendritic tree of granule cells was identical in both control cells (*n* = 18) and TrkC-deficient cells (*n* = 19). The number of the primary dendrites was 3.5 (±0.25) in granule cells that were transfected with the shRNA control plasmid vs. 3.8 (±0.43) in cells transfected with the shRNA TrkC plasmid. Similarly, the length of the distal dendrites was more than 50 μm in both cell cultures. There were no significant differences in these measures between control cell population and cells with inhibited activity of TrkC (Figure 9E).

## Discussion

Here, we report that molecular markers for different cerebellar cell types are similar in marsupials and eutherian mammals; however, the lack of TrkC signaling leads to unexpected effects in opossums. Specifically, we found that in newborn opossums, several brain structures, including the cerebellum, are at an early stage of development. Proliferation of cerebellar Purkinje cells started on the second day after birth and lasted until P5. Another cell type that forms the DCN also proliferates during this period. During the following 2 weeks, the cerebellum expands its territories due to growing cells and folding processes. Additionally, glial cell genesis (astrocytes and oligodendrocytes) began on P16 in the cerebellum of opossums. The first granule cells also appeared on this day, and their proliferation lasted for quite a long time. In 3-month-old opossums, the proliferation of a small number of cells in the EGL still persists. Downregulation of TrkC activity increases the number of first-order dendrites in cerebellar Purkinje cells without influencing the ramification of dendrites in cerebellar granule cells.

### Development of the Cerebellum

Although the cellular structure of the cerebellum is similar in all mammalian species, there are differences in the relative size of the cerebellum (Sultan and Glickstein, 2007). Similarly, various species are born at different stages of development of the cerebellum. Sánchez-Villagra and Sultan (2002) investigated the developmental stage of the cerebellum in 23 eutherian species, most of which were rodents. They described only cerebellar cortical layers at birth and found that different eutherian species are characterized by a large diversity in the developmental stage of the cerebellum. Among the investigated species, *Sorex araneus* had the most immature cerebellum, whereas species that were born with open eyes had the most mature cerebellum. They also had limited access to *M. domestica* specimens and reported that the cerebellum was immature in 6-day-old opossums. Using Nissl staining, Laxson and King (1983) described the development of the cerebellum in another marsupial species, *Didelphis virginiana*. They noted that the cerebellum was immature at birth, the EGL was visible 5 days after birth, and the first Purkinje cells were present on P12. In this opossum, the development of the cerebellum and its maturation lasted for 77 days. This is comparable to the rat cerebellum on P25 (Korneliussen, 1968). These data were published in the early 1980s, when methods for studying the development of brain structures were limited.

Over the past several years, numerous studies have identified molecular markers that stain specific cerebellar cells (Bulfone et al., 1995; Ben-Arie et al., 1997; Wang et al., 2005; Morales and Hatten, 2006; Ju et al., 2016). CB, which is a reliable marker for Purkinje cells, used to examine Purkinje cells in several eutherian species, including rodents and primates (Garcia-Segura et al., 1984; Sahin and Hockfield, 1990; Frantz and Tobin, 1994; Fortin et al., 1998; Flace et al., 2014). Our findings demonstrate that Purkinje cells in *Monodelphis* opossums also express CB. In addition, birthdating experiments with BrdU injections showed that the proliferation of Purkinje cells began at birth. In newborn opossums that were injected with BrdU, double labeling for BrdU and CB confirmed the generation of Purkinje cells. This process continues until P5, which is longer than that in mice. In mice, proliferation of Purkinje cells occurs for 2 days, from E10.5 to E12.5 (Hashimoto and Mikoshiba, 2003). Our results demonstrate that neurons in the DCN are also produced during this period. There are two types of neurons in the DCN in rodents: glutamatergic and GABAergic neurons. Glutamatergic neurons in the DCN are the main cells that send information outside the cerebellum (Voogd, 2014). During development, neurons in the DCN express Pax6, *Tbr1*, and *Olig2* (Fink et al., 2006; Seto et al., 2014; Ju et al., 2016). *Olig2* belongs to the family of bHLH transcription factors and is involved in the regulation of cell lineage specification and differentiation. It was first described to be involved in oligodendrocyte differentiation (Lu et al., 2000; Zhou et al., 2000). Later, Takebayashi et al. (2002) reported that Olig2 is involved in neuronal development. According to our data, Tbr1 and Olig2 proteins are transiently produced in neurons of the DCN. The expression patterns of both Tbr1 and Olig2 transcription factors are similar during early embryonic stages. On P11, they are strongly expressed in the DCN. Unlike mouse (Ju et al., 2016), we did not detect the expression of Olig2 in Purkinje cells.

In the opossum, the generation of Purkinje cells lasts approximately 5 days, with very few cells being generated on the last day (P5). At this period, progenitors derived from the VZ migrate and form a second germinal zone, the EGL. Although the first progenitors that create the EGL were generated on P5, these cells were not active before P16, as shown in our birthdating experiments using BrdU injections. Progenitor cells in the EGL of opossums started to proliferate within P16 and P90, generating a huge number of granule cells. During this period of development, Bergmann glial fibers were also clearly immunostained for GFAP. In opossums, the EGL disappears when animals are approximately 5 months old, whereas in mice, granule cells are produced within 2 weeks (Komuro et al., 2001; Marzban et al., 2014).

### Effects of TrkC Receptor Expression on the Morphology of Different Cerebellar Cells

Neurotrophins have a significant effect on morphology of neurons in the developing brain. BDNF and NT3, and their high-affinity receptors TrkB and TrkC, respectively, are essential for cerebellar neuron development. *In vitro* cell culture experiments show that young granule cells respond to BDNF, while mature granule cells respond to NT3 (Segal et al., 1995). This study also revealed that BDNF is necessary for granule cell axon growth. Furthermore, Morrison and Mason (1998) examined the influence of neurotrophins on the development of Purkinje cells using the granule–Purkinje cell co-culture technique. They found that purified Purkinje cells from the mouse cerebellum did not develop dendritic trees when cultured alone. The addition of BDNF to these cell cultures improved the survival of Purkinje cells without any influence on the dendritic tree. The formation of dendritic trees in Purkinje cells was observed only when Purkinje cells were cultured with granule cells in a BDNF environment.

TrkB and TrkC receptors are structurally similar; however, each of these receptors controls different aspects of the development of cerebellar cells. *In vivo* studies have shown that TrkC receptors regulate dendritic growth in mouse Purkinje cells through the activation of the NT3/TrkC signaling pathway (Joo et al., 2014). NT3 mRNA is expressed in cerebellar granule cells but not in Purkinje cells. Purkinje cells express specific NT3 ligand receptors, TrkC (Lindholm et al., 1998), and receive their main afferent inputs from the axons of granule cells. Thus, NT3 in granule cells can affect the development of Purkinje cells through presynaptic axons. This influence of granule cells on the development of Purkinje cell was demonstrated in the following experiments. When NT3 was removed from the granule cells of the cerebellum in mice, the dendritic morphology of Purkinje cells was altered and the number of dendritic branches decreased (Joo et al., 2014). In contrast, our data show that inhibition of TrkC receptor activity in Purkinje cells leads to an increase in the number of dendrites in these cells. However, downregulation of TrkC in granule cells did not alter the characteristic dendritic morphology of these cells. Primary cell cultures of Purkinje cells were prepared using cerebellar progenitor cells from 3-day-old opossums, while primary cell cultures of granule cells were prepared using cerebellar progenitor cells from P22 opossums. Indeed, there was no cerebellar granule cell among the cells cultured on P3; however, Purkinje cells developed dendritic trees during the subsequent days in cell cultures. Furthermore, we observed that the lack of TrkC receptors resulted in increased numbers of dendrites in Purkinje cells. This is an unexpected result because TrkC receptors are involved in neuronal survival and differentiation as well as neurite enhancement (Tessarollo et al., 1993). However, Trk receptors have diverse functions including the activation of opposing signaling pathways leading to both the survival and death of neurons. As shown in mice, the development of Purkinje cells, including generation, differentiation, and maturation, occurs at different stages (for review see Hatten and Heintz, 1995). The opossum is a marsupial characterized by prolonged postnatal development of the cerebellum compared to eutherian mammal species of the same size. We suggest that in opossums, during the early stage of Purkinje cell development, which persists for a long period of time compared to that in mice, Purkinje cells do not need to develop an expanded dendritic tree, particularly when granule cell axons are absent.

Another explanation of these data is that the compensatory mechanism could be activated and resulted in dendritic tree development. Our cell cultures were harvested from the primordial cerebellar tissue that contains the ventricular zone with different types of progenitor cells. Although Purkinje cells were grown *in vitro* under artificial conditions, the medium allowed to maintain normal growth of neuronal cells. Therefore, Purkinje cells were cultured together with many other neurons, most of which express NT3. TrkC deficiency may lead to activation of the neurotrophic receptor p75^NTR^. This receptor regulates many aspects in the developing brain such as cell survival, neurite outgrowth, and, paradoxically, apoptosis, the opposite function that induces cell death (Bibel et al., 1999; Naska et al., 2010; Meier et al., 2019). We found that p75^NTR^ is expressed in the early development of the opossum cerebellum. Most likely, inhibition of the high-affinity TrkC receptor for NT3 enables binding NT3 to p75^NTR^ and exhibits the NT3/p75^NTR^ signaling pathway. Once NT3 binds to p75^NTR^, it acts to regulate dendritic development of Purkinje cells.

In this study, we provide evidence that the generation of cerebellar cells in the opossum starts after birth in newborn opossums. Additionally, intensive progenitor cell proliferation in the EGL lasts for 3 months. Afterwards, the proliferation rate in the EGL was slow; however, proliferation continued until the opossums were 5 months old. Molecular markers such as CB for Purkinje cells, Tbr1 and Olig2 for DCN neurons and oligodendrocytes, NeuN for granule cells, GFAP for Bergmann glial cells, and astrocytes are excellent markers for investigating developmental events in the cerebellums of opossums. Further, we found that the downregulation of TrkC activity increases the number of first-order dendrites, suggesting a new function of TrkC receptors during dendrite arborization.

## Data Availability Statement

The raw data would be available on request to corresponding author.

## Ethics Statement

The animal study was reviewed and approved by the Local Ethics Committee in Warsaw.

## Author Contributions

BT, KB, MO, SN, and MB performed experiments. BT, KB, and KT supplied the acquisition of data and were responsible for the article intellectual content. RD provided the conception, design, and writing of the study.

## Conflict of Interest

The authors declare that the research was conducted in the absence of any commercial or financial relationships that could be construed as a potential conflict of interest.
