# Corrigendum: Downregulation of TrkC Receptors Increases Dendritic Arborization of Purkinje Cells in the Developing Cerebellum of the Opossum, *Monodelphis domestica*

**DOI:** 10.3389/fnana.2020.614617

**Published:** 2020-11-06

**Authors:** Beata Tepper, Katarzyna Bartkowska, Malgorzata Okrasa, Sonia Ngati, Magdalena Braszak, Kris Turlejski, Ruzanna Djavadian

**Affiliations:** ^1^Laboratory of Calcium Binding Proteins, Nencki Institute of Experimental Biology Polish Academy of Sciences, Warsaw, Poland; ^2^Faculty of Biology and Environmental Sciences, Cardinal Stefan Wyszynski University in Warsaw, Warsaw, Poland

**Keywords:** development, cerebellum, TrkC receptor, shRNA, BrdU, *Monodelphis*, opossum

In the published article, there was an error in the superscript number that appeared after the corresponding author's name. Instead of “**2**”, it should be “**1**”.

The authors apologize for this error and state that this does not change the scientific conclusions of the article in any way. The original article has been updated.

