# Supplementary figures and images for: Downregulation of TrkC Receptors Increases Dendritic Arborization of Purkinje Cells in the Developing Cerebellum of the Opossum, Monodelphis domestica
Source: Front Neuroanat. 2020 Sep 10;14:56. doi: 10.3389/fnana.2020.00056 (PMC7511753; doi:10.3389/fnana.2020.00056)

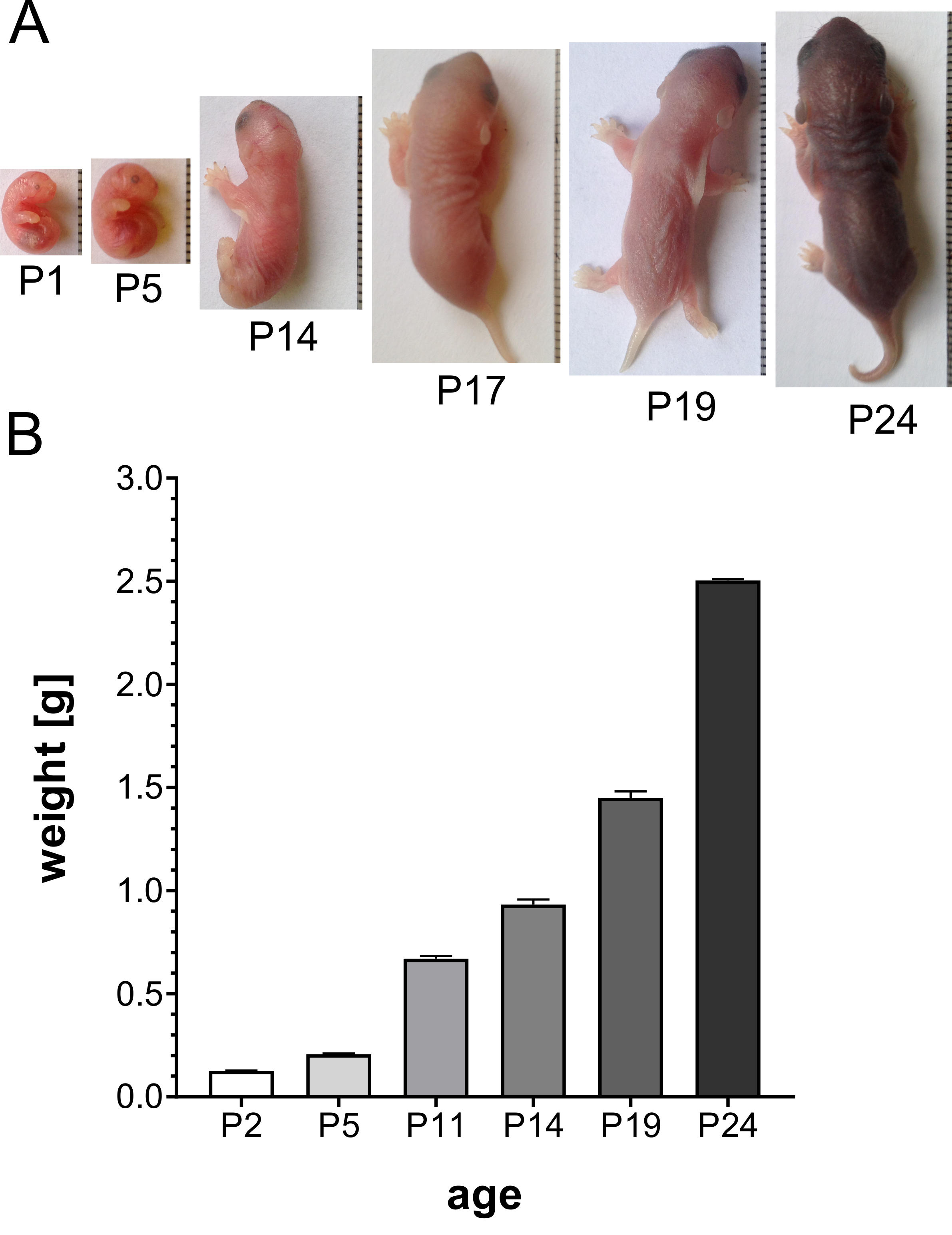

Supplement: Supplementary file 1 [file Image_1.TIF]
